# Novel *MYH11::GLI3* fusion in ileal leiomyoma

**DOI:** 10.3389/pore.2026.1612375

**Published:** 2026-04-13

**Authors:** Ioannis Panagopoulos, Ingvild Lobmaier

**Affiliations:** 1 Section for Cancer Cytogenetics, Institute for Cancer Genetics and Informatics, Oslo University Hospital-Radiumhospitalet, Oslo, Norway; 2 Department of Molecular Oncology, Institute for Cancer Research, Oslo University Hospital-Radiumhospitalet, Oslo, Norway; 3 Department of Pathology, Oslo University Hospital-Radiumhospitalet, Oslo, Norway

**Keywords:** fusion gene, gastrointestinal tract, ileum, leiomyoma, *MYH11::GLI3*

## Abstract

**Background:**

Leiomyomas of the gastrointestinal tract (GI) are benign smooth muscle neoplasms with limited genetic characterization. Molecular investigations may improve diagnostic classification and enhance understanding of their biological behavior.

**Methods:**

RNA sequencing using multiple fusion-detection algorithms was performed on an ileal leiomyoma. Key findings were validated by RT-PCR and Sanger sequencing.

**Results:**

A *MYH11::GLI3* fusion was identified. Additional chimeric transcripts were detected but interpreted as secondary events based on limited read support. The biological relevance of *MYH11::GLI3* relates to smooth muscle specific *MYH11* expression and *GLI3*-mediated Hedgehog signaling.

**Conclusion:**

This study reports, for the first time, the identification of a MYH11::GLI3 chimera in gastrointestinal leiomyoma, thereby expanding the molecular spectrum of these tumors. Deregulation of GLI3 may represent an alternative mechanism of Hedgehog pathway perturbation in this neoplasm. The frequency and clinical significance of GLI3-rearranged gastrointestinal smooth muscle tumors remain to be determined.

## Introduction

Mesenchymal tumors of the gastrointestinal (GI) tract comprise a diverse group of neoplasms that are classified according to their cell of origin or line of differentiation into several categories, including adipocytic, fibroblastic/myofibroblastic, neurogenic, myogenic, vascular/perivascular, and tumors of uncertain differentiation [1–3]. Based on their biological behavior, these tumors are further subdivided into benign, intermediate (locally aggressive or rarely metastasizing), and malignant types [1–3].

The most common mesenchymal neoplasm of the GI tract is the gastrointestinal stromal tumor (GIST), which is thought to originate from, or show differentiation toward, the interstitial cells of Cajal [1–3]. Approximately 80% of GISTs harbor activating mutations in the KIT proto-oncogene receptor tyrosine kinase, located on chromosome 4q12, while about 10% carry mutations in the platelet-derived growth factor receptor alpha (*PDGFRA*), also located on 4q12 [4, 5]. Cytogenetically, GISTs frequently show chromosomal aberrations, most commonly involving losses of chromosome arms 14q, 22q, 1p, and 15q [6]. Immunohistochemically, GISTs typically show strong expression of KIT (CD117), ANO1 (DOG-1), and CD34 [4, 5]. A small subset of GISTs harbor oncogenic fusion genes involving *BRAF*, *FGFR1*, and *NTRK3* [7, 8]

Leiomyomas are the second most common GI mesenchymal tumors and are typically found in the esophagus, stomach, small intestine, and colon [1, 2, 9]. Histologically, leiomyomas closely resemble normal smooth muscle cells, and immunohistochemically they express DES (desmin), ACTA2 (α-SMA), CALD1 (caldesmon 1), and CNN1 (calponin 1) [1, 2]. In esophageal and gastric leiomyomas, scattered tumor cells may additionally express KIT and ANO1 [10]. Genetic studies of GI leiomyomas are limited. A deletion involving the *COL4A5/COL4A6* locus on chromosome Xq22 has been reported in an esophageal leiomyoma [11], genomic imbalances have been detected in three cases [12], and an *FN1::ALK* fusion gene has been identified in two GI leiomyomas [13]. Because recurrent fusion genes have been identified in uterine and extra-uterine leiomyomas, including gastrointestinal leiomyomas, we performed RNA sequencing to investigate whether a fusion gene or other transcript-level alterations were present in the current tumor.

In the present study, we describe a leiomyoma of the ileum harboring a novel *MYH11::GLI3* fusion gene, thereby expanding the molecular spectrum of GI smooth-muscle tumors and providing additional insight into their genetic heterogeneity.

## Methods

Total RNA was extracted from tumor tissue stored at −80 °C using the miRNeasy kit (Qiagen, Hilden, Germany) and submitted to the Genomics Core Facility, Norwegian Radium Hospital, Oslo University Hospital, for high-throughput paired-end RNA sequencing. Fusion transcripts were identified using the FusionCatcher, Arriba, and STAR-Fusion algorithms [14–16].

Complementary DNA (cDNA) was synthesized from 400 ng of total RNA using the iScript Advanced cDNA Synthesis Kit for RT-qPCR (Bio-Rad Laboratories, Hercules, CA, USA). cDNA corresponding to 20 ng of input RNA was used as template for subsequent PCR amplifications with Premix Taq (Takara Bio Europe/SAS, Saint-Germain-en-Laye, France). PCR was performed using the primers MYH11-2F1 (5′-AGA​TTT​GGA​CGC​TCC​GGC​CTG-3′) and GLI3-899R (5′-AGC​GAT​GGG​CTG​CTG​TGC​AAG-3′). The amplified cDNA fragment was subsequently sequenced using the BigDye Direct Cycle Sequencing Kit (Thermo Fisher Scientific, Waltham, MA, USA) with the primers M13For-MYH11-21F1 (5′-TGT​AAA​ACG​ACG​GCC​AGT​TGG​GAG​GTG​CGT​CAG​ATC​CGA-3′) and M13Rev-GLI3-821R1 (5′-CAG​GAA​ACA​GCT​ATG​ACC​CTC​GGA​AGC​AGC​AGT​GGG​GTT​C-3′). Sequence data were analyzed using BLAST against the NCBI reference sequences NM_002474.3 (*MYH11*) and NM_000168.6 (*GLI3*), and genomic alignment was performed using BLAT and the UCSC Genome Browser with the GRCh38/hg38 human genome assembly [17, 18]. Sequence data have been deposited in GenBank under accession numbers PX926336-PX926343.

## Results

**FIGURE 1:**
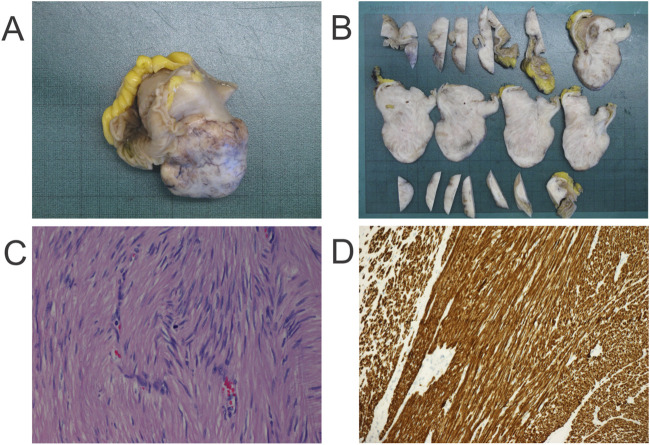
Pathological features of the ileal tumor. **(A)** Operative specimen. **(B)** Gross appearance showing a well-circumscribed tumor with a firm, white, whorled cut surface. **(C)** Histological section showing intersecting fascicles of elongated spindle cells with blunt-ended, cigar-shaped nuclei and abundant eosinophilic cytoplasm; a single mitotic figure is visible (hematoxylin and eosin stain, ×400). **(D)** Immunohistochemical staining for desmin showing strong cytoplasmic positivity (×200).

*Case presentation:* A 45-year-old man underwent abdominal computed tomography (CT) because of abdominal discomfort, which revealed an 8 cm tumor localized to the ileum. The tumor was surgically excised without prior biopsy (Figure 1A). On macroscopic examination, the lesion was well circumscribed and showed a white, firm, whorled cut surface (Figure 1B). Histological examination demonstrated a spindle cell neoplasm composed of long intersecting fascicles of elongated cells with blunt-ended, cigar-shaped nuclei and abundant eosinophilic cytoplasm, consistent with a smooth muscle tumor (Figure 1C). Mitotic activity was very low, with only one mitotic figure identified, and no tumor necrosis was observed. Immunohistochemical analysis showed strong positivity for desmin (Figure 1D), h-caldesmon, and smooth muscle actin, while CD117 and DOG1 were negative. As part of the diagnostic workup, G-banding and karyotypic analysis of metaphase spreads revealed the following karyotype: 44–45,XY,der(1)t(1; 7)(p31; q11),-7,der(16)t(7; 16)(p13∼14; p13)[cp10]/46,XY [2]. The tumor was diagnosed as an ileal leiomyoma.

**FIGURE 2:**
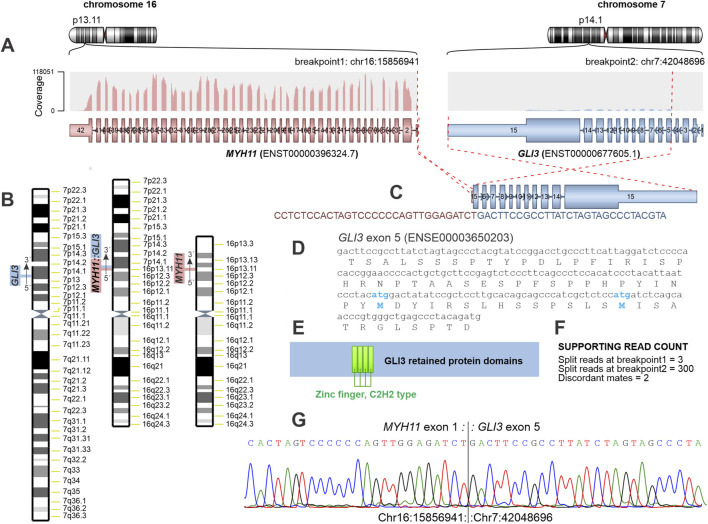
Identification and validation of the *MYH11::GLI3* fusion in an ileal leiomyoma. **(A)** Schematic representation of the *MYH11::GLI3* fusion detected by RNA sequencing, showing fusion of *MYH11* exon 1 with *GLI3* exon 5. **(B)** Chromosome ideograms illustrating the inferred der(16)t(7; 16) translocation giving rise to the *MYH11::GLI3* fusion; the normal chromosomes 7 and 16 are shown for comparison. **(C)** Nucleotide sequence spanning the *MYH11::GLI3* fusion junction. **(D)** Nucleotide sequence of *GLI3* exon 5 and the corresponding predicted amino-acid sequence. Internal ATG codons and the encoded methionine residues (M) are highlighted in blue and bold. **(E)** Schematic representation of the GLI3 protein, indicating retained functional domains in the predicted fusion product. The entire shaded (blue) region represents the retained portion of GLI3, corresponding to amino-acid residues 201–1,580 of the reference sequence (NP_000159.3). **(F)** Read support for the *MYH11::GLI3* fusion as detected by the Arriba fusion-detection algorithm. **(G)** RT-PCR amplification and Sanger sequencing confirming the junction between *MYH11* exon 1 and *GLI3* exon 5.

Analysis of RNA-sequencing data using the three fusion-detection algorithms FusionCatcher, Arriba, and STAR-Fusion identified two fusion genes, *MYH11::GLI3* and *USP48::TSPAN2*, both detected by all three algorithms (Table 1; Figure 2; Supplementary Figure S1; Supplementary Figure S2). In addition, two further fusion genes, *TSPAN2::URGCP* and *SUCO::RABGAP1L*, were detected by FusionCatcher and Arriba but were not retained in the final STAR-Fusion output (Table 1; Supplementary Figure S3; Supplementary Figure S4). RT-PCR and Sanger sequencing confirmed the *MYH11::GLI3* fusion, demonstrating fusion of *MYH11* exon 1 to *GLI3* exon 5 (Figure 2). No additional chimeric transcripts were investigated.

**TABLE 1:** Fusion transcripts identified by RNA sequencing using three fusion-detection algorithms in an ileal leiomyoma. Junction-crossing read counts are shown for each fusion-detection algorithm. Fusions with low read support or detected by a subset of callers were interpreted as secondary events.

Fusion gene	5′- partner fusion point	3′- partner fusion point	Junction-crossing reads			Fusion sequence	GenBank accession number
			FusionCatcher	Arriba	STAR-fusion		
*MYH11::GLI3*	16:15856941:-	7:42048696:-	31	303	1,114	CGA​GCT​CGC​CAT​CCA​GTT​TCC​TCT​CCA​CTA​GTC​CCC​CCA​GTT​GGA​GAT​CT::GAC​TTC​CGC​CTT​ATC​TAG​TAG​CCC​TAC​GTA​TCC​GGA​CCT​GCC​CTT​CAT​TA	PX926336
	16:15856941:-	7:42048765:-	4	Not detected	Not detected	CGA​GCT​CGC​CAT​CCA​GTT​TCC​TCT​CCA​CTA​GTC​CCC​CCA​GTT​GGA​GAT​CT::AGA​CAG​CCT​CTG​CCT​GTG​GAG​ATA​TTT​GTC​TCA​TGC​ATA​CCC​CTT​GTA​TC	PX926337
	16:15856941:-	7:42040239:-	2	1	Not detected	GCC​ATC​CAG​TTT​CCT​CTC​CAC​TAG​TCC​CCC​CAG​TTG​GAG​ATC​T::GCA​CCA​GAT​TCT​CCA​GCC​CCA​GGC​TGT​CAG​CCA​GGC​CGA​GCC​G	PX926338
*USP48::TSPAN2*	1:21695066:-	1:115073007:-	18	12	19	TCT​CGT​TTC​TGC​TAA​TCA​GAC​GTT​AAA​AGA​ATT​GAA​AAT​TCA​G::CTG​GCT​GGA​TCG​GCC​GTC​ATT​GCT​TTT​GGA​CTA​TGG​TTT​CGG​T	PX926339
*TSPAN2::URGCP*	1:115089364:-	7:43887816:-	6	3	Not detected	GTG​CAT​CAA​GTA​CCT​GCT​GCT​TGG​CTT​CAA​CCT​GCT​CTT​CTG​G::GAT​AGA​AGT​GGA​ATT​ACT​GGG​CAA​AGG​GCA​TTC​AGA​TTT​GGG​A	PX926340
	1:115089364:-	7:43887485:-	Not detected	1	Not detected	GCG​GTG​CAT​CAA​GTA​CCT​GCT​GCT​TGG​CTT​CAA​CCT​GCT​CTT​CTG​G::GCA​TTC​AGA​TTT​GGG​AGA​AGT​AGC​CCC​AGA​AAT​AAA​AGC​ATC​AGA​G	PX926341
*SUCO::RABGAP1L*	1:172591071:+	1:174370979:+	2	1	Not detected	AAT​CGT​GAA​ACT​TCA​GAA​TAC​TTC​AAG​AAT​AGC​AGA​GGA​GCA​G::AGA​GTG​ATA​ATG​AAC​TCT​CAA​GTG​GAA​CAG​GTG​ATG​TGT​CTA​A	PX926342
	1:172533497:+	1:174393995:+	Not detected	1	Not detected	CGG​CGG​GCC​TTG​GCC​CTG​GTC​TCC​TGC​CTC​TTT​CTG​TGC​TCT​CTG​GTC​TG::GCA​CAG​TAA​CCT​TGG​TGC​ACG​ACC​GAA​AGG​GCT​GTC​TAC​TCT​GGT​GAA​GA	PX926343

## Discussion

To our knowledge, this study is the first report of *GLI3* rearrangement and formation of a *MYH11::GLI3* fusion in an enteric leiomyoma, thereby expanding the molecular spectrum of these neoplasms. The predicted fusion structure suggests that regulatory sequences from *MYH11* may drive aberrant expression of *GLI3*, potentially leading to dysregulated transcriptional activity.

The *MYH11::GLI3* fusion is consistent with involvement of the derivative chromosome der(16)t(7; 16)(p13∼14; p13). Both *MYH11* and *GLI3*, located at chromosome bands 16p13 and 7p14, respectively, are transcribed in a centromere-to-telomere orientation. Accordingly, the *MYH11::GLI3* chimera is most likely located on the aberrant chromosome der(16) (Figure 2).

The additional chimeric genes (*USP48::TSPAN2*, *TSPAN2::URGCP*, and *SUCO::RABGAP1L*) are compatible with rearrangements involving chromosome 1, consistent with the presence of the derivative chromosome der(1)t(1; 7)(p31; q11) identified by chromosome banding analysis. In particular, involvement of *USP48* (1p36.2), *TSPAN2* (1p13.2), and *URGCP* (7p13) indicates that multiple breakpoints involving chromosome arms 1p and 7p have occurred in the generation of the derivative chromosome der(1)t(1; 7).

These additional chimeric genes were regarded as secondary or passenger events, arising in the context of underlying chromosomal complexity rather than representing biologically driving alterations. This interpretation was based on their low read support across fusion-detection algorithms and restricted detection to a subset of fusion callers (Table 1).

In contrast, the *MYH11::GLI3* fusion is of particular biological relevance. This is supported by its high read counts across fusion-detection algorithms (Table 1; Figure 2), the involvement of *MYH11* as a 5′ fusion partner, and the well-established role of *GLI3* as a transcriptional regulator with dosage-sensitive biological effects [19].

The *MYH11* gene encodes smooth muscle myosin heavy chain and is a well-established marker of smooth muscle differentiation [20]. Its expression is driven by a promoter that is among the most specific and tightly regulated in differentiated smooth muscle cells [21]. *MYH11* rearrangements are best known from the chromosomal aberrations inv(16)(p13q22) and t(16; 16) in acute myeloid leukemia, resulting in the *CBFB::MYH11* fusion [22, 23]. *MYH11* has only rarely been implicated in solid tumors [24]. Its involvement as the 5′ fusion partner in the present case is consistent with the smooth-muscle phenotype of the tumor and suggests that *MYH11* may contribute regulatory elements that drive aberrant expression of the fusion partner.

The *GLI3* gene (7p14.1), together with *GLI1* (12q13.3), *GLI2* (2q14.2), and *GLI4* (8q24.3), encodes the members of the GLI family of zinc-finger transcription factors [25]. These transcription factors bind to the consensus DNA sequence 5′-GACCACCCA-3′ in the promoters of target genes, regulate their transcriptional activity, and act as transcriptional mediators of the Hedgehog signaling pathway [26–29]. Aberrant activation of Hedgehog signaling has been implicated in the initiation and progression of multiple cancer types and contributes to diverse aspects of tumorigenesis [30–34].

*GLI3* is unique among members of the GLI family in that it can function either as a transcriptional activator or as a transcriptional repressor, depending on cellular context and Hedgehog pathway activity [19, 35, 36]. In the presence of Hedgehog signaling, full-length GLI3 accumulates and acts as a transcriptional activator, commonly referred to as GLI3A, thereby promoting expression of downstream target genes [19, 35]. In the absence of Hedgehog signaling, full-length GLI3 undergoes proteolytic processing to generate a truncated repressor form, designated GLI3R, which translocates to the nucleus and suppresses transcription of Hedgehog target genes [19, 36, 37].

The main *MYH11::GLI3* fusion transcript, detected by all three fusion-detection algorithms, joins the untranslated exon 1 of *MYH11* to exon 5 of *GLI3*. As a consequence, *GLI3* exons 1-4, including the canonical translation initiation codon (ATG) located in exon 2, are absent from the chimeric transcript. However, exon 5 of *GLI3*, which is retained in the fusion, contains internal ATG codons that may serve as alternative translation initiation sites (Figure 2). An alternative *MYH11::GLI3* fusion transcript, detected by FusionCatcher and Arriba, joins exon 1 of *MYH11* to exon 7 of *GLI3*, which likewise contains internal ATG codons that may function as alternative translation initiation sites (Table 1; Supplementary Figure S1). Translation from these internal start codons would be predicted to generate an N-terminally truncated GLI3 protein retaining the DNA-binding zinc-finger domain and downstream C-terminal functional domains. Based on the predicted fusion structure, the retained region would correspond to amino-acid residues 201–1,580 of the GLI3 reference protein (NP_000159.3), or residues 309–1,580 in the alternative fusion transcript. Importantly, expression of the *MYH11::GLI3* fusion transcript would be driven by the highly specific and tightly regulated *MYH11* promoter, potentially resulting in lineage-restricted, aberrant expression of truncated *GLI3* in smooth muscle cells. Support for the plausibility of this mechanism comes from experimental evidence demonstrating that *GLI3* can be translated from non-canonical start sites [38]. In a CRISPR-Cas9 study targeting the endogenous *GLI3* gene, cells carrying biallelic out-of-frame mutations were nevertheless found to express GLI3 protein, despite disruption of the canonical reading frame [38]. The authors attributed this unexpected protein expression to illegitimate translation, likely initiated from internal or non-canonical start codons downstream of the mutations. These findings indicate that *GLI3* is permissive to alternative translation initiation and can give rise to truncated but stable protein products [38]. In the context of the present *MYH11::GLI3* fusion, a similar mechanism may operate, whereby internal ATG codons within *GLI3* exon 5 or exon 7 serve as alternative translation initiation sites, resulting in expression of an N-terminally truncated GLI3 protein retaining key functional domains (Figure 2; Supplementary Figure S1). Although protein-level validation was not feasible in the present case, the transcript structure and prior experimental evidence support the biological plausibility of this mechanism. Such non-canonical translation mechanisms are increasingly recognized in cancer and developmental contexts, where alternative start codon usage and truncated protein isoforms may contribute to oncogenic signaling diversity [39–43].

Recently, *GLI1*-enteric tumors have been proposed as a distinct subgroup within *GLI*-altered neoplasms, separable from other tumor types, particularly myoepithelial tumors of soft tissue and glomus tumors [44, 45]. These tumors generally follow an indolent clinical course, but may carry an increased risk of aggressive behavior when exceeding 5 cm in size or exhibiting high-grade morphology [44, 45]. *MALAT1::GLI1* and *ACTB::GLI1* represent the most frequently identified fusion genes in *GLI1*-enteric tumors; however, these fusions are not disease-defining, as they are also observed in plexiform fibromyxoma and gastroblastoma [44, 45].

The identification of a *MYH11::GLI3* fusion in the present ileal leiomyoma suggests that deregulation of *GLI3* represents an alternative mechanism of Hedgehog pathway perturbation in enteric tumors and, more broadly, in gastrointestinal smooth-muscle neoplasms. Given that *MYH11* expression is driven by a promoter that is highly specific and tightly regulated in differentiated smooth muscle cells, the *MYH11::GLI3* fusion may result in lineage-restricted, aberrant expression of *GLI3* in smooth-muscle cells. Whether this fusion alters the balance between GLI3 activator and repressor functions, or instead leads to ectopic or deregulated *GLI3* expression driven by the *MYH11* promoter and independent of canonical Hedgehog pathway regulation, remains to be determined.

From a diagnostic perspective, gastrointestinal smooth muscle tumors that do not fully meet established criteria for leiomyoma or leiomyosarcoma remain challenging entities. As illustrated by the present case, genetic investigations may contribute to improved diagnostic classification and a better understanding of biological behavior. In selected cases, identification of a *MYH11::GLI3* fusion may serve as a molecular marker of smooth muscle differentiation and help define a genetically distinct subset of enteric tumors. The apparent rarity of *GLI3* rearrangements may reflect biological constraints and tissue-specific detection bias, rather than true absence. More broadly, the identification of recurrent or characteristic fusion genes may, in the future, help refine the classification of gastrointestinal smooth muscle tumors and distinguish biologically distinct subsets within this heterogeneous group.

## Conclusion

The present case expands the spectrum of fusion genes identified in gastrointestinal smooth muscle tumors and highlights *MYH11::GLI3* as a novel fusion gene in this setting. Further studies are warranted to determine the frequency of *GLI3* rearrangements, identify potential alternative 5′ fusion partners, and clarify their biological and clinical significance in gastrointestinal smooth muscle tumors.

## Data Availability

The datasets presented in this study can be found in online repositories. The names of the repository/repositories and accession number(s) can be found in the article/Supplementary Material.
