# Supplementary material for: Novel MYH11::GLI3 fusion in ileal leiomyoma
Source: Pathol Oncol Res. 2026 Apr 13;32:1612375. doi: 10.3389/pore.2026.1612375 (PMC13111170; doi:10.3389/pore.2026.1612375)
Supplement: Supplementary file 1 [file DataSheet1.pdf]

Supplementary Figure legends

Supplementary Figure S1. Schematic representation of low-abundance alternative *MYH11::GLI3* fusion transcript detected by RNA sequencing. RNA sequencing revealed a low-abundance *MYH11::GLI3* fusion transcript in the ileal leiomyoma, detected by FusionCatcher and Arriba but not by STAR-Fusion. The fusion involves *MYH11* on chromosome 16p13.11 (transcript ENST00000396324.7) and *GLI3* on chromosome 7p14.1 (transcript ENST00000677605.1). Genomic breakpoints were mapped to chr16:15,856,941 within *MYH11* and chr7:42,040,239 within *GLI3*, consistent with a t(7;16) rearrangement. Exon-intron structures and read coverage profiles are shown, with introns not drawn to scale. The fusion joins *MYH11* exon 1 to *GLI3* exon 7, resulting in loss of the N-terminal region of *GLI3*. Nucleotide sequence of *GLI3* exon 7 (ENSE00003684714) and the corresponding predicted amino-acid sequence are shown. The internal ATG codon and the encoded methionine residue (M) are highlighted in blue and bold. The fusion preserves the zinc-finger (C2H2-type) DNA-binding domains of GLI3. The entire shaded (blue) region represents the retained portion of GLI3, corresponding to amino-acid residues 277-1580 of the reference sequence (NP_000159.3). Supporting RNA-sequencing evidence is limited and consists of a very low number of split reads at the fusion junction, as indicated. The lower panels illustrate the inferred fusion transcript architecture and retained GLI3 protein domains.

Supplementary Figure S2. Schematic representation of low-abundance *USP48::TSPAN2* fusion transcript detected in the ileal leiomyoma. RNA sequencing identified a fusion between *USP48* on chromosome 1p36.12 (transcript ENST00000529637.5) and *TSPAN2* on chromosome 1p13.2 (transcript ENST00000369516.7), with genomic breakpoints at chr1:21,695,066 and chr1:115,073,007, respectively. Exon-intron structures and read coverage profiles are shown, with introns not drawn to scale. The fusion is predicted to be in-frame and retains the ubiquitin carboxyl-terminal hydrolase domain of USP48 and the tetraspanin domain of TSPAN2. Supporting RNA-sequencing evidence consists of a limited number of split reads at both fusion junctions and few discordant mate pairs, indicating low-level expression. Given the low supporting read counts, this fusion was not considered a primary genetic event but rather interpreted as a secondary or passenger fusion arising in the context of underlying chromosomal complexity.

Supplementary Figure S3. Schematic representation of low-abundance *TSPAN2::URGCP* fusion transcripts detected in the ileal leiomyoma. RNA sequencing identified two closely related, low-abundance *TSPAN2::URGCP* fusion transcripts involving *TSPAN2* on chromosome 1p13.2 (transcript ENST00000369516.7) and *URGCP* on chromosome 7p13 (transcript ENST00000453200.6). The shared 5′

breakpoint is located at chr1:115,089,364 within *TSPAN2*, while alternative 3′ breakpoints map to chr7:43,887,816 (panel A; *URGCP* exon 2) and chr7:43,887,485 (panel B; *URGCP* exon 3). Exon-intron structures and read coverage profiles are shown, with introns not drawn to scale. Both fusion transcripts are predicted to be out-of-frame and retain the tetraspanin family domain of TSPAN2; the URGCP portion encompasses most of the coding sequence, with loss of only a few N-terminal amino acids. Supporting RNA-sequencing evidence is limited and consists of a small number of split reads and discordant mate pairs. The fusion shown in panel A was detected by both FusionCatcher and Arriba, whereas the fusion shown in panel B was detected by Arriba only; neither fusion transcript was detected by STAR-Fusion. Given the low supporting read counts and limited concordance across fusion-calling algorithms, these fusion transcripts were not considered primary genetic events but were interpreted as secondary or passenger fusions arising in the context of underlying chromosomal complexity.

Supplementary Figure S4. Schematic representation of low-abundance *SUCO::RABGAP1L* fusion transcripts detected in the ileal leiomyoma. RNA sequencing identified two low-abundance *SUCO::RABGAP1L* fusion transcripts involving SUCO located on chromosome 1q24.3 (transcript ENST00000367723.8) and RABGAP1L on chromosome 1q25.1 (transcript ENST00000681986.1). The fusion transcripts are generated by alternative breakpoint combinations, with genomic breakpoints mapping to chr1:172,591,071 and chr1:174,370,979 (panel A) and to chr1:172,533,497 and chr1:174,393,995 (panel B). Exon-intron structures and read coverage profiles are shown, with introns not drawn to scale. The fusion shown in panel A *(SUCO* exon 2::*RABGAP1L* exon 13) is predicted to be in-frame and retains the Rab-GTPase-TBC domain of RABGAP1L. The fusion shown in panel B (*SUCO* exon 18::*RABGAP1L* exon 12) is predicted to be out-of-frame and to disrupt the coding sequence; however, it includes the C-terminal Sad1/UNC-like domain of SUCO as part of the chimeric transcript. Supporting RNA-sequencing evidence is very limited and consists of a single split read at the *RABGAP1L* breakpoint for each junction, with no discordant mate pairs detected, indicating extremely low-level expression. The fusion shown in panel A was detected by Arriba only, whereas the fusion shown in panel B was detected by both FusionCatcher and Arriba; neither fusion transcript was detected by STAR-Fusion. Given the low supporting read counts and limited concordance across fusion-calling algorithms, these fusion transcripts were not considered primary genetic events but were interpreted as secondary or passenger fusions arising in the context of underlying chromosomal complexity.

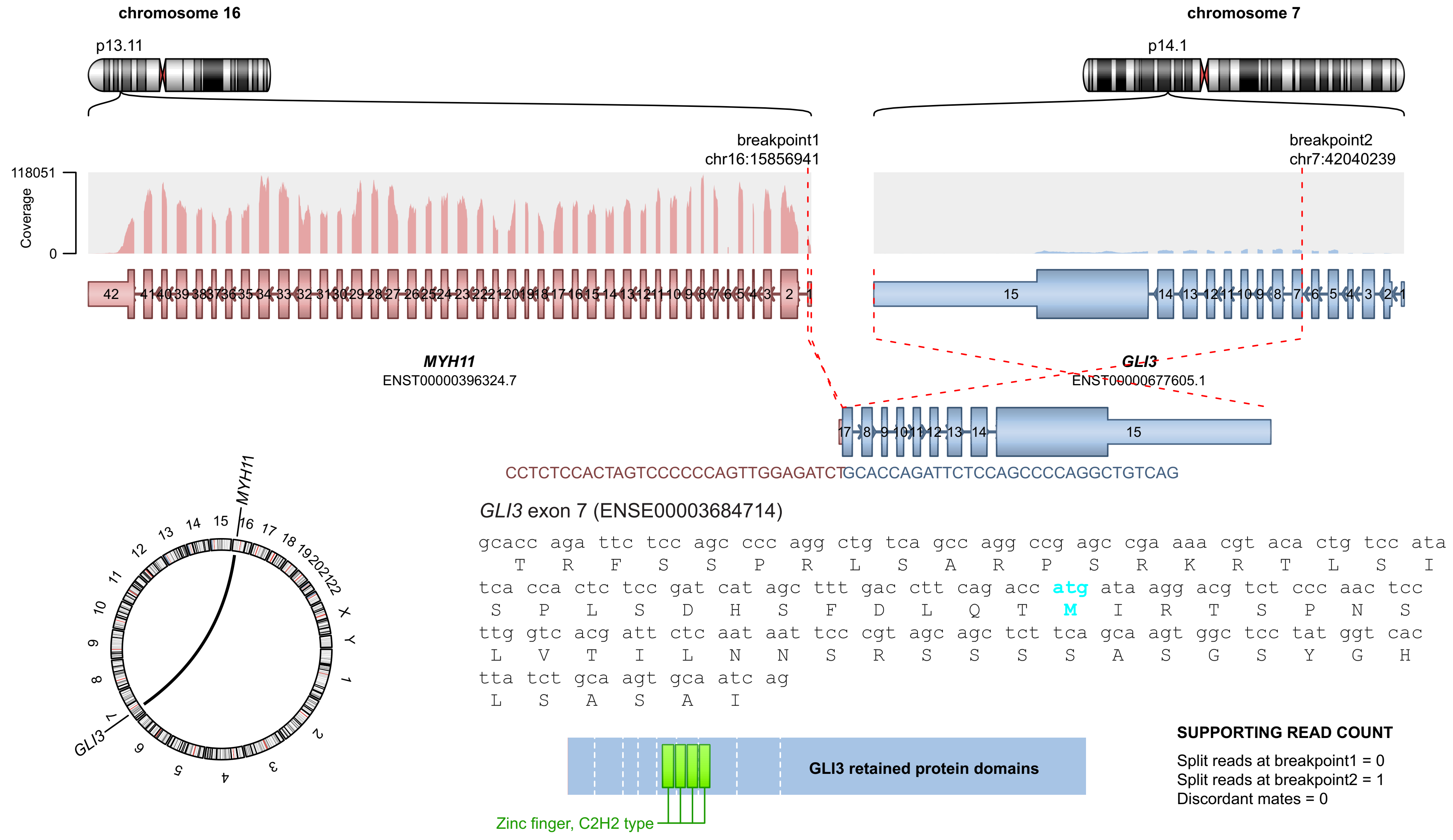

Supplementary Figure S1. Schematic representation of low-abundance alternative *MYH11::GLI3* fusion transcript detected by RNA sequencing.

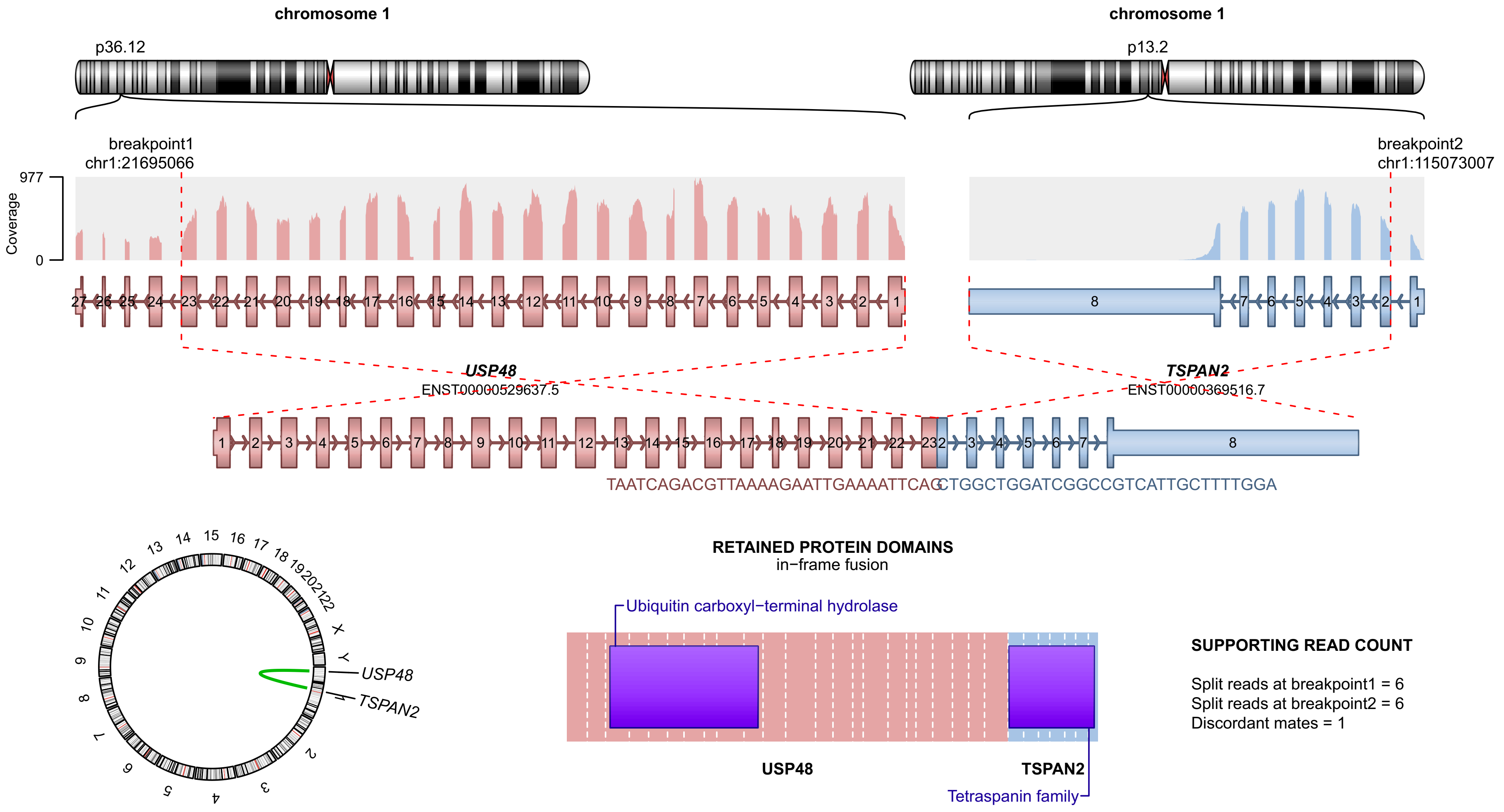

Supplementary Figure S2. Schematic representation of low-abundance *USP48::TSPAN2* fusion transcript detected in the ileal leiomyoma.

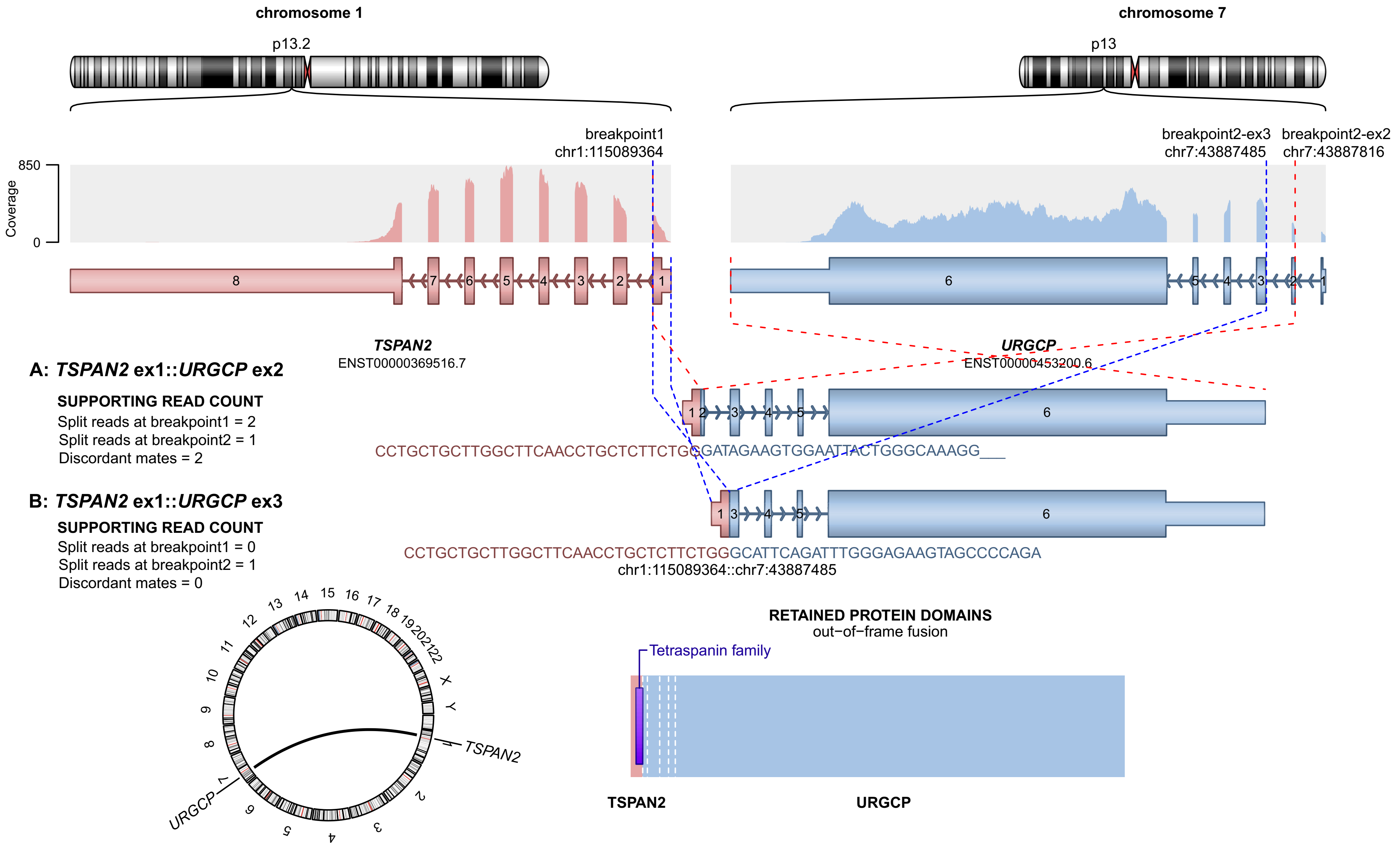

Supplementary Figure S3. Schematic representation of low-abundance *TSPAN2::URGCP* fusion transcripts detected in the ileal leiomyoma.

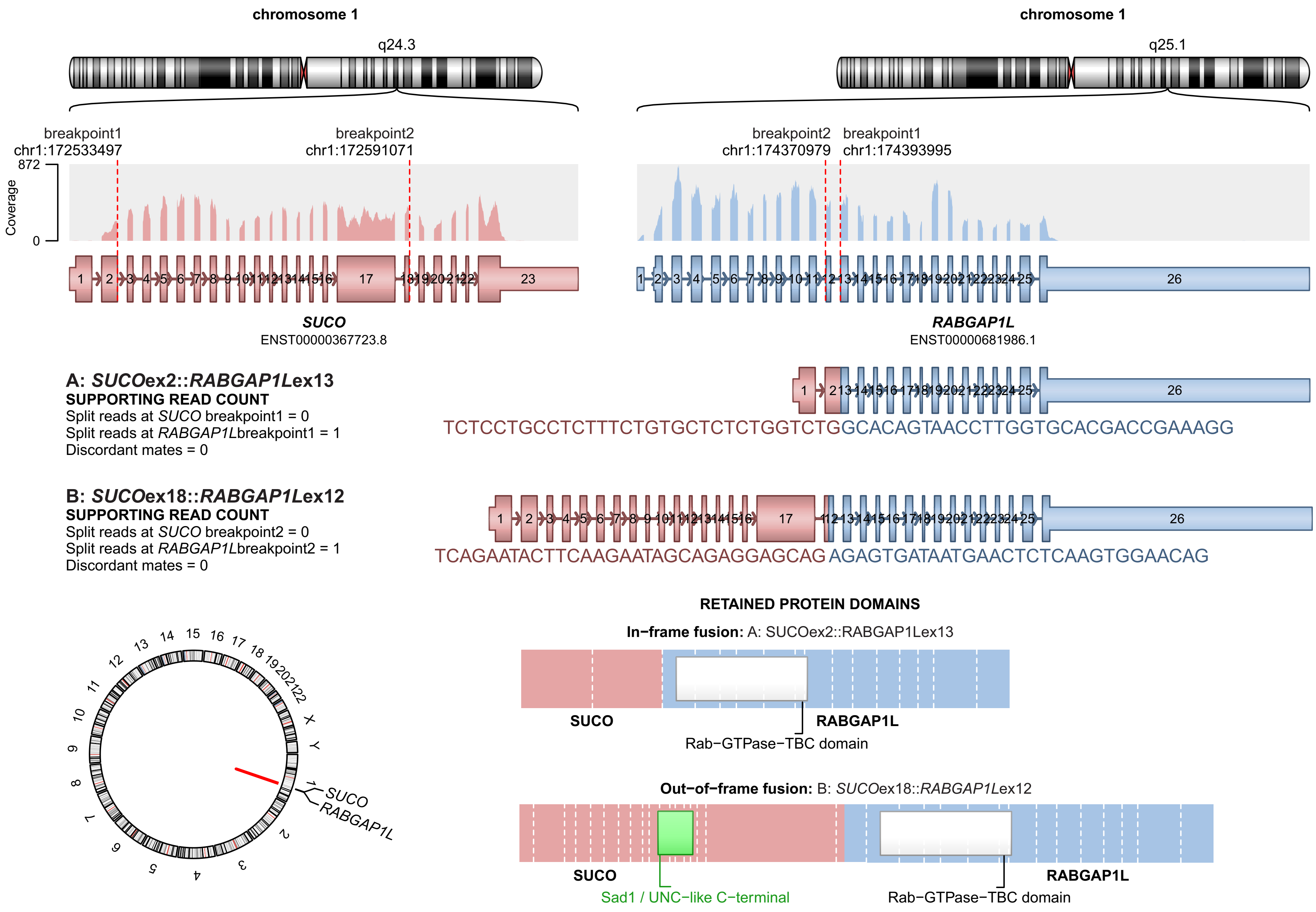

Supplementary Figure S4. Schematic representation of low-abundance *SUCO::RABGAP1L* fusion transcripts detected in the ileal leiomyoma.
